# Retinal blood vessel diameters in children and adults exposed to a simulated altitude of 3,000 m

**DOI:** 10.3389/fphys.2023.1026987

**Published:** 2023-02-28

**Authors:** Tinkara Mlinar, Tadej Debevec, Jernej Kapus, Peter Najdenov, Adam C. McDonnell, Anton Ušaj, Igor B. Mekjavic, Polona Jaki Mekjavic

**Affiliations:** ^1^ Department of Automatics, Biocybernetics and Robotics, Jozef Stefan Institute, Ljubljana, Slovenia; ^2^ Jozef Stefan International Postgraduate School, Ljubljana, Slovenia; ^3^ Faculty of Sport, University of Ljubljana, Ljubljana, Slovenia; ^4^ Department of Paediatrics, General Hospital Jesenice, Jesenice, Slovenia; ^5^ Department of Biomedical Physiology and Kinesiology, Simon Fraser University, Burnaby, BC, Canada; ^6^ Eye Hospital, University Medical Centre, Ljubljana, Slovenia; ^7^ Faculty of Medicine, University of Ljubljana, Ljubljana, Slovenia

**Keywords:** hypoxia, children, adults, central retinal arteriolar equivalent, central retinal venular equivalents

## Abstract

**Introduction:** Technological advances have made high-altitude ski slopes easily accessible to skiers of all ages. However, research on the effects of hypoxia experienced during excursions to such altitudes on physiological systems, including the ocular system, in children is scarce. Retinal vessels are embryologically of the same origin as vessels in the brain, and have similar anatomical and physiological characteristics. Thus, any hypoxia-related changes in the morphology of the former may reflect the status of the latter.

**Objective:** To compare the effect of one-day hypoxic exposure, equivalent to the elevation of high-altitude ski resorts in North America and Europe (∼3,000 m), on retinal vessel diameter between adults and children.

**Methods:** 11 adults (age: 40.1 ± 4.1 years) and 8 children (age: 9.3 ± 1.3 years) took part in the study. They spent 3 days at the Olympic Sports Centre Planica (Slovenia; altitude: 940 m). During days 1 and 2 they were exposed to normoxia (F_i_O_2_ = 0.209), and day 3 to normobaric hypoxia (F_i_O_2_ = 0.162 ± 0.03). Digital high-resolution retinal fundus photographs were obtained in normoxia (Day 2) and hypoxia (Day 3). Central retinal arteriolar equivalent (CRAE) and venular equivalents (CRVE) were determined using an Automated Retinal Image Analyser.

**Results:** Central retinal arteriolar and venular equivalents increased with hypoxia in children (central retinal arteriolar equivalent: 105.32 ± 7.72 µm, hypoxia: 110.13 ± 7.16 µm, central retinal venular equivalent: normoxia: 123.39 ± 8.34 µm, hypoxia: 130.11 ± 8.54 µm) and adults (central retinal arteriolar equivalent: normoxia: 105.35 ± 10.67 µm, hypoxia: 110.77 ± 8.36 µm; central retinal venular equivalent: normoxia: 126.89 ± 7.24 µm, hypoxia: 132.03 ± 9.72 µm), with no main effect of group or group*condition interaction. A main effect of condition on central retinal arteriolar and venular equivalents was observed (central retinal arteriolar equivalent:normoxia: 105.34 ± 9.30 µm, hypoxia: 110.50 ± 7.67 µm, *p* < 0.001; central retinal venular equivalent: normoxia: 125.41 ± 7.70 µm, hypoxia: 131.22 ± 9.05 µm, *p* < 0.001).

**Conclusion:** A 20-hour hypoxic exposure significantly increased central retinal arteriolar and venular equivalents in adults and children. These hypoxia-induced increases were not significantly different between the age groups, confirming that vasomotor sensitivity of the retinal vessels to acute hypoxia is comparable between adults and prepubertal children.

## 1 Introduction

Skiing is a popular family winter activity, to which children are introduced at a young (prepubertal) age. Technological developments (chairlifts and cable cars) have made many high-altitude (≥3,000 m; please see Table 1) ski slopes easily accessible to all levels of skiers, including children (Meijer and Jean, 2008; Giesenhagen et al., 2019). At these altitudes, several environmental factors, such as cold, ultraviolet radiation, and hypoxia, may significantly impact not only physical performance (Cymerman et al., 1996; Jaki Mekjavic et al., 2021), but other physiological and psychological systems as well. Indeed, impairment of the aerobic performance, resulting from the lower partial pressure of oxygen (PO_2_) at altitude (Wehrlin and Hallén, 2006), has been observed in both adults and children, with the magnitude of such being similar (Kriemler et al., 2016; Kapus et al., 2017). The present study investigated the effect of hypoxia on retinal vessels, which share similar anatomical, physiological, and embryological characteristics with cerebral vessels (Baker et al., 2008). Thus, any changes observed in the retinal vasculature may reflect similar processes occurring within the brain vasculature.

**TABLE 1:** Characteristics of high-altitude (peak altitude: ≥3,000 m) ski resorts in North America (SKIRESORT.INFO, 2022b) and Europe (SKIRESORT.INFO, 2022a).

	Europe	North America
Number of ski resorts (peak altitude: ≥3,000 m)	45	49
Minimum base altitude (m)	768	1924
Maximum base altitude (m)	2,760	3,290
Maximum peak altitude (m)	3,899	3,914
Maximum *Δ* altitude in one resort (m)	2,807	1,340

For a list of all 94 ski resorts, please see Supplementary Table (Abbreviations: *Δ* altitude, the difference between the peak and base altitude).

The ocular apparatus is a commonly affected system at altitude (Jaki Mekjavic et al., 2021). As a consequence of the retina’s high oxygen demand as highly metabolically active tissue (Eshaq et al., 2014), adequate retinal oxygenation is essential for the maintenance of normal visual function. To match the increased retinal oxygen demand during hypoxic exposure, retinal vessels dilate and become more tortuous to allow for the required increases in blood flow (Neumann et al., 2016). Supplementary to vasodilation of the vessels, other retinal changes such as cotton wool spots, haemorrhages, and papilledema, commonly known under the umbrella term high altitude retinopathy (HAR), may be observed upon ascent to altitude (Morris et al., 2006). The formation of these symptoms may be further exacerbated by strenuous exercise, such as skiing (Honigman et al., 2001) or mountaineering (Shults and Swan, 1975), and the examination of these features in prepubertal children is lacking.

To ensure safety at altitude, particularly during activities such as skiing, it is essential to determine the effect of hypoxia on the visual system in different age groups. Therefore, within the framework of the KidSki project (Kapus et al., 2017; Ušaj et al., 2019), we set out to investigate the effect of a one-day exposure to hypoxia, equivalent to the elevation of high-altitude ski resorts in North America and Europe (∼3,000 m), on the retinal blood vessels, specifically the arterioles and venules, between adults and children.

## 2 Materials and methods

### 2.1 Study design

**FIGURE 1:**
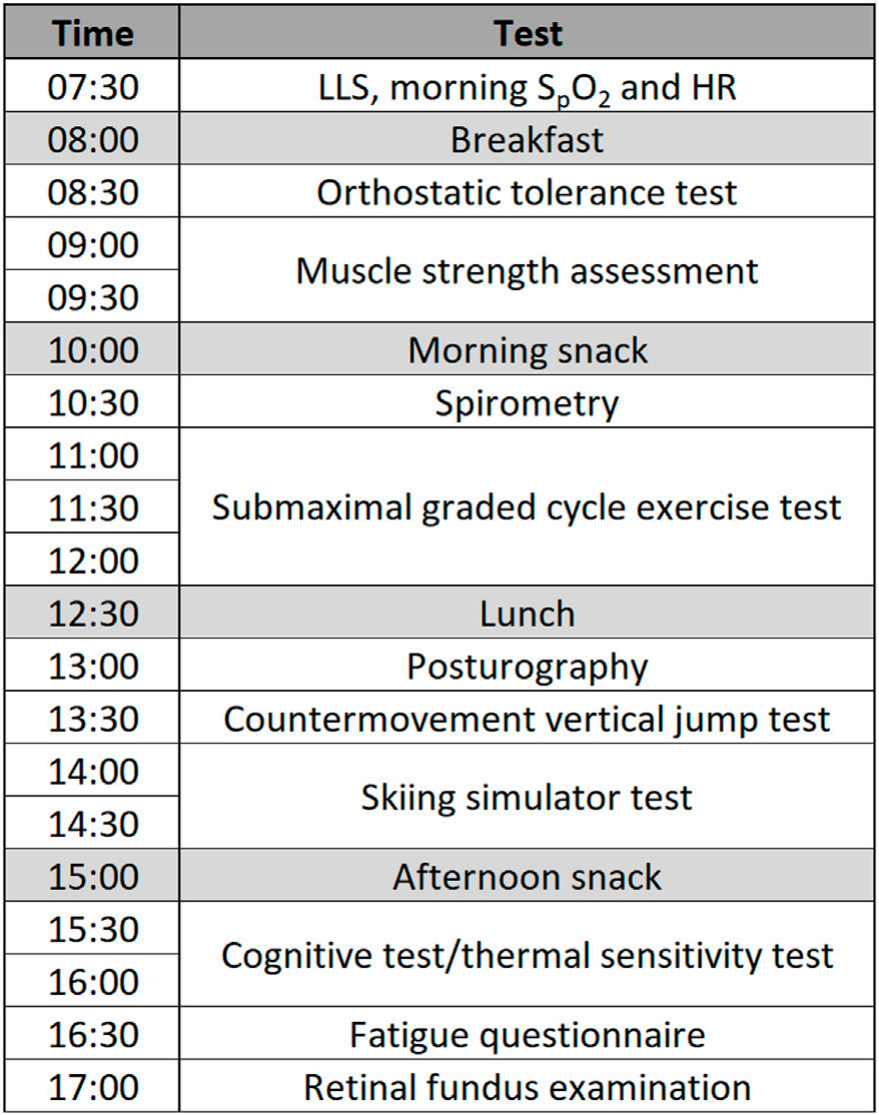
Testing schedule for Day 1 (normoxia) and Day 2 (hypoxia) (Note: LLS, Lake Louise score; S_p_O_2_, peripheral oxygen saturation; HR, heart rate).

Details of the study protocol have been outlined previously (Kapus et al., 2017). Briefly, each participating family (parents and children) visited the Olympic Sports Centre Planica (Rateče, Slovenia) on one occasion for 3 days. On Day 1, participants arrived at the facility in the afternoon and were familiarised with the researchers, laboratory, equipment, and experimental procedures. They also underwent a medical examination and spent the first (Day 1) and second day (Day 2) in normoxia [altitude of the facility: 940 m; F_i_O_2_ = 20.9%; P_i_O_2_ = 134.0 ± 0.4 mmHg]. The participants entered the normobaric hypoxic environment at 20:00 on the second night where they remained throughout the night and Day 3 until the termination of the experimental procedure. By reducing the oxygen content of the air, the normobaric hypoxic environment simulated an equivalent altitude of 3,000 m [F_i_O_2_ = 0.162 ± 0.03; P_i_O_2_ = 105.0 ± 0.6 mmHg]. Throughout Day 2 and Day 3 the participants took part in a series of physiological tests (for further details *cf.* Kapus et al., 2017). The experimental procedures on Day 2 (normoxia) and Day 3 (hypoxia) were conducted in the same order and at the same time of the day to avoid any diurnal fluctuations in the measured variables. The testing schedule (Figure 1) was designed in a way that the constraints for each test were met, and limited interaction was present between the tests. A paediatrician was present throughout the study.

The normobaric hypoxic conditions were established with a vacuum-pressure swing adsorption system (VPSA), which delivered hypoxic gas to all the rooms and laboratory. The system sampled the gas in all rooms at 15-min intervals to ensure that the pre-set level of oxygen fraction was being maintained. In the event that the oxygen fraction decreased below the pre-set in any of the rooms, the system would cease the delivery of hypoxic gas to that room. Should the oxygen fraction not return to the desired level within two sampling cycles, the system would automatically initiate a fan that would deliver external air to the room, and also trigger an alarm. All rooms were equipped with portable clip-on type oxygen sensors programmed to initiate an alarm, should the oxygen fraction decrease below a pre-set threshold. There were no such untoward events during the course of the study.

### 2.2 Participants

In total, 13 adults (7 males, 6 females) and 13 children (Tanner stage 1; 7 males, 6 females) took part in the KidSki project. Children’s participation in the study was subject to the consent of the parents, who also participated in the study, and upon the approval of the paediatrician, who conducted the medical examination. Due to the specifications of the Automated Retinal Image Analyser (ARIA; Peter Bankhead, Queen’s University Belfast) software, only retinal scans of 11 adults (7 males, 4 females) and 8 children (4 males, 4 females) were used in the final analysis. All participants were lowland residents with no hypoxic exposure in the 2 months prior to taking part in the study as specified in the inclusion criteria. Participants’ physical characteristics are presented in Table 2.

**TABLE 2:** Participants’ physical characteristics.

	Children	Adults
Number (M/F)	8 (4/4)	11 (7/4)
Age (years)	9.3 ± 1.3	40.1 ± 4.1
Height (cm)	141.2 ± 11.2	176.0 ± 8.5
Weight (kg)	31.2 ± 7.5	72.9 ± 12.1
BMI (kg·m^-2^)	15.4 ± 1.6	23.4 ± 2.2
BF% (%)	10.8 ± 5.9	20.2 ± 9.2

Note: M, males; F, females; BMI, body mass index; BF%, body fat percentage.

Exclusion criteria included smoking (adults only), asthma, hypertension, haematological or kidney disorders, exposure to altitude (>2,500 m) in the preceding 2 months, and any eye condition that could influence retinal vessels. Throughout the study, adult participants were requested not to consume any caffeine or alcohol.

The study conformed to the standards set by the Declaration of Helsinki, except for registration in a database. Prior to taking part in the study, the participants were thoroughly informed about the aims, methodology, and potential risks of the study, after which signed consent forms were obtained from the adults, and the children gave their vocal consent. The children’s consent forms were subsequently signed by the children and final consent was obtained from their parents/guardians, who were also participants in the study. The study was approved (approval no. 164/05/13) by the National Medical Ethics Committee at the Ministry of Health (Republic of Slovenia).

### 2.3 General acclimation to hypoxia

The participants’ resting morning heart rate (HR) and oxygen saturation (S_p_O_2_) were measured in normoxia (Day 2) and hypoxia (Day 3) upon waking using a finger pulse oximeter (Nellcor, BCI 3301, Boulder, United States). Additionally, to assess for the potential presence of acute mountain sickness (AMS), participants completed the self-assessment section of the Lake Louise mountain sickness questionnaire (Roach et al., 1993) to obtain the Lake Louise score (LLS; 0–15). The collection of children’s LLS was adapted following the recommendations of the International Federation for Climbing and Mountaineering Medical Commission guidelines (Meijer and Jean, 2012).

### 2.4 Retinal fundus examination

Digital high-resolution retinal colour fundus photographs of the left and right eyes centred on the optic disc were taken according to procedures described elsewhere (De Boever et al., 2014). Measurements were performed by an ophthalmologist using a 45° 6.3 megapixel digital non-mydriatic camera (Canon, Hospithera, Brussels, Belgium). During the fundus photography, patients were seated on a chair in a darkened room with their chin resting on a chin support. The presence of HAR was determined with direct fundus ophthalmoscopy.

### 2.5 Data processing and analysis

Retinal vessels were analysed so that left and right eye central retinal arteriolar (CRAE) and venular equivalent (CRVE) in hypoxia and normoxia were determined using the ARIA. The calibration was set at 8.077 µm per pixel. Only retinal scans where the same three venules and three arterioles within the region between 0.5- and 1.0-disc diameters away from the disc margin were able to be identified in both normoxia and hypoxia, were used in the statistical analysis. In the event that an obtained retinal scan was of poor quality, the scan was repeated. Upon completion of the study, all scans were analysed with ARIA. The image analysis software deemed some of the scans of insufficient quality to be used in the analysis. Since the aim of the study was a comparison of images obtained before and after the normoxic and hypoxic confinements, a poor-quality scan obtained either pre- or post-exposure for a given participant, would render the results of this participant unusable by the software, requiring exclusion of the participant from the analysis. As a consequence, only retinal scans of 8 children (4 females) and 11 adults (4 females) were of quality that could be properly analysed by ARIA. Due to the high inter-eye correlation reported previously (Leung et al., 2003a), the average value of the left and right eyes was used in the statistical analysis. The eye examinations were performed by an ophthalmologist, thus any changes of clinical concern would have been identified immediately.

All of the statistical analyses were performed using SPSS (v.25, IBM, NY, United States) software. The data are presented as mean ± SD unless indicated otherwise. The significance level for all statistical tests in this study was set at *p* < 0.05, *a priori*. Unbiased effect sizes were estimated using Hedges’ *g* test and defined as small when *g* ≤ 0.2, moderate when *g* ≤ 0.5, and large when *g* ≤ 0.8 (Hedges, 1981). All data were assessed for normality using the Shapiro-Wilk test of normality. A paired-samples *t*-test was used to assess whether the effect of hypoxia (hypoxia vs. normoxia) was present in the morning HR and S_p_O_2_ measurements, and Wilcoxon signed-rank test to assess the effect of hypoxia in LLS. Potential hypoxia-induced CRAE and CRVE differences between both age groups were investigated using a mixed model ANOVA [group (children, adults)*condition (hypoxia, normoxia)] and potential changes between CRAE and CRVE within each age group were assessed using a two-way repeated measures ANOVA [vessel (CRAE, CRVE)*condition (hypoxia, normoxia)]. Where appropriate, a Bonferroni *post hoc* test was applied to investigate interaction effects in greater detail.

## 3 Results

No negative events related to the hypoxic environment were reported by the participants, nor were they observed by the attending paediatrician.

### 3.1 General acclimation to hypoxia

Morning resting HR was significantly higher in hypoxia compared to normoxia in children [*t*(7) = −3.108, *g* = 0.74*, p* = 0.017] but not in adults [*t*(10) = −0.359, *p* = 0.727], as seen in Table 3.

**TABLE 3:** Adults’ and children’s morning HR, S_p_O_2_ and LLS following a 12-hour night-time hypoxia exposure.

	Children		Adults	
Condition	Normoxia	Hypoxia	Normoxia	Hypoxia
Heart rate (min^-1^)	80.1 ± 15.4	92.5 ± 16.1*	64.2 ± 14.8	65.5 ± 13.0
S_p_O_2_ (%)	98.0 ± 1.4	92.4 ± 1.7*	96.5 ± 0.8	90.1 ± 2.7*
LLS (median (range))	0 (0–2)	1.5 (0–4)	0 (0–2)	1 (0–4)

Note: M, males; F, females; S_p_O_2_, peripheral oxygen saturation; LLS, Lake Louise score; * significantly different than in normoxia; *p* ≤ 0.05.

Morning resting S_p_O_2_ decreased significantly in hypoxia in both age groups (children: *t*(7) = 6.840, g = 3.42, *p* < 0.001; adults: *t*(10) = 8.061, *g* = 3.07, *p* < 0.001). There was no statistical difference in S_p_O_2_ between the two groups.

Hypoxia had no significant effect on children’s (*Z* = −1.897, *p* = 0.058) or adults’ (*Z* = −1.549, *p* = 0.121) LLS. LLS values ≥ 3 were observed in two adults and two children following a 12-hour night-time exposure to hypoxia. The median (range) LLS values in hypoxia were comparable between the two age groups. No other signs of AMS were observed by the attending paediatrician.

### 3.2 Retinal fundus

#### 3.2.1 High altitude retinopathy

Based on direct fundus ophthalmoscopy, the ophthalmologist confirmed that no signs of HAR were present.

#### 3.2.2 Central retinal arteriolar equivalent

**FIGURE 2:**
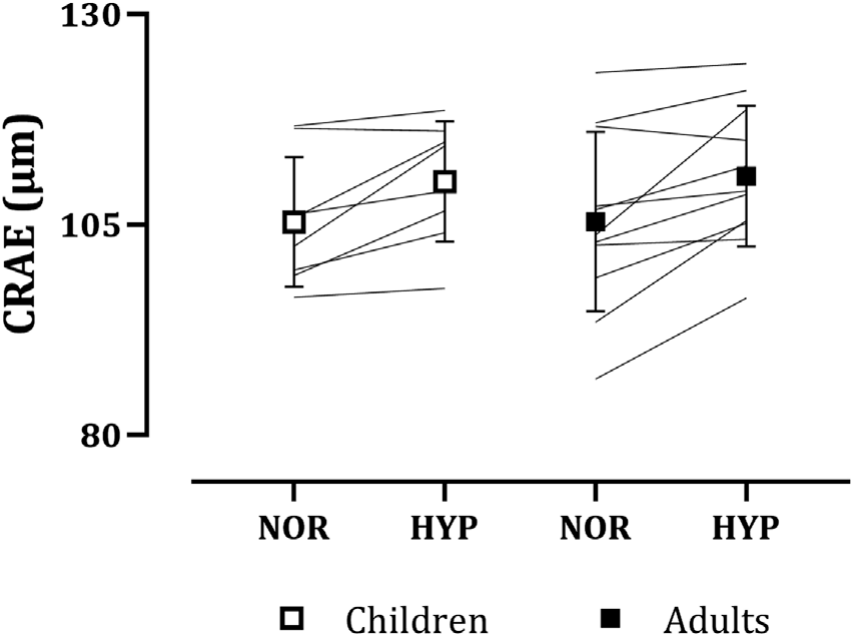
Adults’ and children’s central retinal arteriolar equivalents (CRAE) in normoxia (NOR) and hypoxia (HYP).

CRAE increased with hypoxia in both children (normoxia: 105.32 ± 7.72 µm, hypoxia: 110.13 ± 7.16 µm) and in adults (normoxia: 105.35 ± 10.67 µm, hypoxia 110.77 ± 8.36 µm), as seen in Figure 2. No main effect of group (*p* = 0.933) or group*condition interaction (*p* = 0.785) was present. In contrast, a main effect of condition on CRAE was observed (normoxia: 105.34 ± 9.30 µm, hypoxia: 110.50 ± 7.67 µm, *p* < 0.001, *g* = 0.59).

#### 3.2.3 Central retinal venular equivalent

**FIGURE 3:**
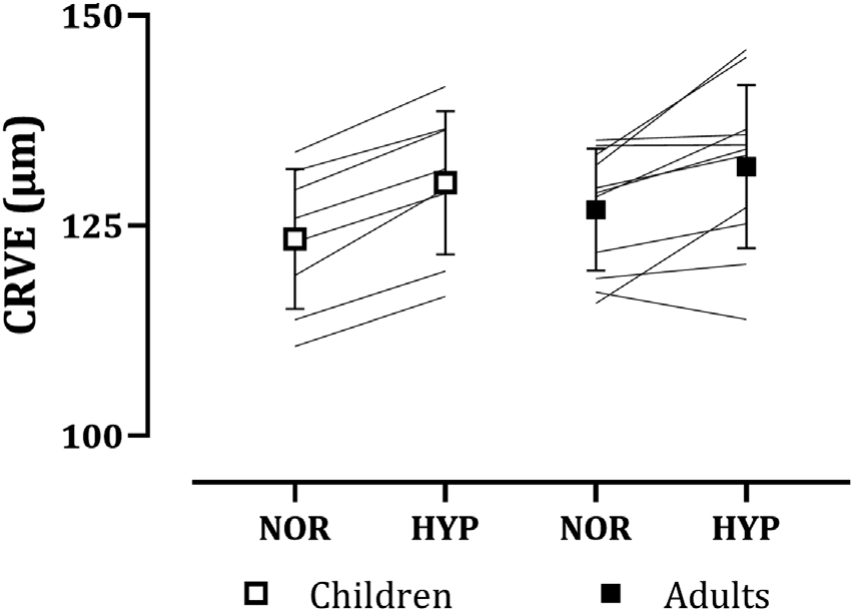
Adults’ and children’s central retinal venular equivalents (CRVE) in normoxia (NOR) and hypoxia (HYP).

Similarly to CRAE, CRVE increased with hypoxia in both children (normoxia: 123.39 ± 8.34 µm, hypoxia: 130.11 ± 8.54 µm) and adults (normoxia: 126.89 ± 7.24 µm, hypoxia: 132.03 ± 9.72 µm), as seen in Figure 3. Again, no main effect of group (*p* = 0.488) or group*condition interaction (*p* = 0.446) was present. A main effect of condition on CRVE was observed (normoxia: 125.41 ± 7.70 µm, hypoxia: 131.22 ± 9.05 µm, *p* < 0.001, *g* = 0.68).

#### 3.2.4 Central retinal arteriolar vs. venular equivalent

A main effect for vessels was observed both in children (CRAE: 107.73 ± 7.61 µm, CRVE: 126.75 ± 8.86 µm, *p* = 0.001, *g* = 2.25) and adults (CRAE: 108.06 ± 9.76 µm, CRVE: 129.46 ± 8.77 µm, *p* < 0.001, *g* = 2.27). However, no main effect of vessel*condition interaction was observed in either age group (children: *p* = 0.218; adults: *p* = 0.878).

## 4 Discussion

The main finding of the present study is that a 20-hour hypoxic exposure equivalent to an altitude of ∼3,000 m caused significant increases in the diameter of retinal venules and arterioles in adults and children. Furthermore, hypoxia-induced increases in CRAE and CRVE were not significantly different between the two age groups. This confirms that the vasomotor sensitivity of the retinal vessels to acute hypoxia is comparable between adults and prepubertal children.

### 4.1 The effect of hypoxia on retinal vessel diameters

Despite the high blood flow, the total blood volume of the retinal vessels is relatively low due to their small diameter and sparse distribution (Wolf et al., 1991). As mentioned previously, the retina is one of the most metabolically active tissues in the body, with a high arteriovenous PO_2_ difference and poor capacity to tolerate periods of low perfusion (Funk, 1997). Both the central retinal artery, which supplies the majority of blood to the retina (Kur et al., 2012), and the vein lack neural innervation which could provide regulation of the vascular tone. Therefore, blood flow in the retina is maintained by autoregulation, that is mainly dependent on local myogenic and metabolic factors, including arterial blood gases, pH, and lactate (Venkataraman et al., 2006; Garhöfer et al., 2003). In case of reduced oxygen availability, as reflected by the reduced S_p_O_2_, adequate retinal blood flow is achieved through vasodilation to match the augmented oxygen demand (Morris et al., 2006; Neumann et al., 2016). In the present study, morning S_p_O_2_ following a 12-hour overnight hypoxic exposure decreased from 97.2% ± 1.2% in normoxia (Day 2) to 91.3% ± 2.4% (both age groups combined), initiating vasodilation in both retinal arterioles and venules. The reactivity of adult retinal vessels to various levels of PO_2_ was first reported by Cusick et al. (1942). Their findings have since been replicated and confirmed during exposure to both acute and chronic hypoxia (Louwies et al., 2016; Neumann et al., 2016), including in the present study. Additionally, retinal changes, including changes in retinal vessel diameter, have previously been observed in both normobaric and hypobaric hypoxia. The effect on the retinal vessel diameter was not dependent on the ambient pressure (i.e., normobaric *versus* hypobaric hypoxia), but solely on PO_2_ (Neumann et al., 2016). No such research has previously been conducted in children.

Arteriolar walls consist of smooth muscle cells, whereas the venular walls are thinner and consist of a single layer of endothelial cells and a few smooth muscle cells (Kur et al., 2012). It has been proposed that venular walls are therefore more compliant and potentially exhibit larger autoregulatory responses (Louwies et al., 2016). The results of several studies investigating the diameters of retinal vessels while breathing hyperoxic or hypoxic gas mixtures are equivocal. While some authors reported greater reactivity of venules than arterioles (Cusick et al., 1942; Louwies et al., 2016), other studies observed no differences in reactivity (Fallon et al., 1985; Neumann et al., 2016), including the present study where the diameter of retinal arterioles increased by 5.16 ± 4.67 µm in hypoxia, and similarly, the diameter of venules increased by 5.81 ± 4.28 µm (both age groups combined).

### 4.2 Adults vs. children

Retinal vessel diameters decrease with age, independent of blood pressure and other factors (Leung et al., 2003b; Wong et al., 2003). On the contrary, neither CRAE nor CRVE of the children and adults participating in the present study were significantly different. Most likely, the adults in the present study were not old enough (40.1 ± 4.1 years) for the age-related retinal changes to be manifest. For example, participants in studies conducted by Leung et al. (2003b); Wong et al. (2003) were aged >49 and >43 years, respectively, whereas the age range of adult participants in our study varied from 32 to 47 years (median: 40 years). Due to the low sample size, correlation analysis of age and CRAE and CRVE was not conducted.

Normoxic retinal vessel diameters measured in children and adults participating in the present study are smaller than the values reported in the literature (Mitchell et al., 2007; Li et al., 2011; Neumann et al., 2016). A similar phenomenon was observed in adults’ CRAE and CRVE measurements during exposure to hypoxia (Neumann et al., 2016). This can primarily be attributed to the differences in the analysis technique. In the present study, only three venules and three arterioles per retinal scan were included in the analysis. Due to the limited region of interest (between 0.5- and 1.0-disc diameters away from the optic disc margin), overlapping of vessels, and their branching, it was not always possible to select the six widest vessels.

Hypoxia-induced increases in retinal vessel diameters observed in both adults and children were not significantly different between the two age groups. To our knowledge, this is the first study to investigate children’s retinal vessel diameter changes during exposure to hypoxia. As discussed previously, hypoxia-induced vasodilation in retinal vessels in adults observed in the present study is in line with the previous research (Cusick et al., 1942; Louwies et al., 2016; Neumann et al., 2016). When looking at adults’ responses, a considerable individual variability can be noted, especially in the adult’s CRVE, compared to the children’s. This is also reflected in the standard deviation of the responses (±1.70 µm in children and ±5.46 µm in adults). Perhaps, such a range in individual responses of CRVE to hypoxia could be partially attributed to a considerable age range of adult participants.

### 4.3 High altitude headache, high altitude retinopathy and acute mountain sickness

HAR and AMS encompass a spectrum of physiological and pathological changes commonly occurring in unacclimatised individuals exposed to hypoxia. High-altitude headache (HAH), a type of headache occurring during ascents to altitudes above 2,500 m and resolving spontaneously within 24 h after descent, can appear along with other signs and symptoms which constitute AMS (Burtscher et al., 2011; Bian et al., 2013) or as an isolated symptom.

HAR has been noted to occur more frequently in individuals undergoing strenuous activity during hypoxic exposure, especially when Valsalva manoeuvres are involved (Arora et al., 2011). Meanwhile, symptoms of AMS are more commonly observed in adults than in children (Rexhaj et al., 2011; Kriemler et al., 2014). Other risk factors for the development of AMS, HAR and/or HAH include the level of hypoxic exposure, the rate and length of ascent, low arterial oxygen saturation, and an individual’s susceptibility (McFadden et al., 1981; Schneider et al., 2002; Bian et al., 2013).

Arteriolar and venular retinal vessel diameter has previously been correlated with the development of AMS (Bosch et al., 2009). Additionally, HAH burden was found to correlate strongly with retinal venous vasodilatation (Wilson et al., 2013).

In the present study, the presence of HAR, AMS, and/or HAH was not observed or reported by any of the participants. This is likely the result of the following two factors: 1) the level of hypoxia was low, and 2) the duration of the exposure was short.

### 4.4 Clinical implications

The retinal and cerebral macro- and microvasculature share many morphological and physiological properties, including similar vascular regulatory processes (Delaey and Van de Voorde, 2000). Due to these similarities, changes in cerebral microvasculature, resulting from diseases such as vascular dementia (Varma et al., 2002) and stroke (Powers and Zazulia, 2003) are often reflected in changes in retinal microvasculature. Furthermore, hypoxia-induced cerebral venous vasodilation has been observed to correlate strongly with the venous vasodilation observed in the retina (Wilson et al., 2013). It has been proposed that the small retinal vessel leakage that occurs in individuals exposed to high altitudes could also be mimicked in the vessels in their brain, contributing to the brain volume increase of a few millilitres observed following hypoxic exposures (Kallenberg et al., 2007; Willmann et al., 2013).

The role of high altitude-induced changes to the retina in predicting the manifestation of a life-threatening high altitude cerebral oedema is not yet fully understood (Barthelmes et al., 2011; Willmann et al., 2014).

Exposure to hypoxia can have a significant effect on human vision, manifesting as changes in colour discrimination (Connolly et al., 2008), reduction in dark adaptation (Kobrick and Appleton, 1971), and loss of contrast sensitivity (Pescosolido et al., 2015), with the changes being more evident in low light environments (Connolly et al., 2008). In contrast, high altitude has no effect on visual acuity (Bosch et al., 2009). In the event of blood leakage into the vitreous humour or when HAR-related haemorrhages manifest on the macula, the result can be an acute severe visual impairment. Most commonly, these hypoxia-induced vision changes are not clinically important and are reversed within weeks upon return to normoxia. Similarly, vasodilation of retinal vessels is reversible when adequate oxygen availability is restored (Bosch et al., 2009; Jaki Mekjavic et al., 2021). Visual function tests were not performed in the present study, however, no apparent hypoxia-related effects on vision were observed or reported by the participants, most likely due to low levels of hypoxia.

### 4.5 Limitations

A major limitation of the present study is a small sample size, especially in the younger group, mainly due to measurement errors when obtaining fundus photographs. Altogether retinal scans from 7 participants were discarded and not used in the final analysis because of blurriness, inappropriate lighting and/or composition (e.g., images not centred on the optic disc, mainly due to the inability of younger children to fix their gaze during the measurement). In future studies, this problem can be minimised or eliminated with more thorough familiarisation of the participants with the experimental equipment and procedures, improved measuring techniques, and taking duplicates or triplicates of each retinal scan.

Another limitation of the present study is that mean arterial pressure (MAP) was not measured during the retinal scans. It has previously been reported that MAP is inversely related to retinal vessel diameter (Wong et al., 2003; Kaushik et al., 2007), thus any hypoxia-induced increment in MAP would have caused a decrease in the retinal vessel diameter. In the present study, exposure to hypoxia resulted in retinal vessel vasodilation in both children and adults. Therefore, it can be speculated that retinal vascular regulation is more strongly affected by hypoxia than by the changes in MAP.

## 5 Conclusion

The present study mimicked a one-day family skiing trip to an altitude equivalent to the elevation of ski resorts in North America and Europe (∼3,000 m). No presence of AMS, HAR or HAH was observed in any of the participants. Significant increases in retinal vessel diameters with hypoxia were observed in both children and adults. The level of hypoxia-induced vasodilation did not differ between the two age groups. Adults and children appear to be similarly sensitive to changes in ambient PO_2_, therefore when travelling to altitude, children should do so with the same precautions as adults. No acute hypoxia-related effects on the ocular system were observed in any of the participants. Since retinal vessel vasodilation on its own does not have any clinical consequences, we conclude that skiing at altitudes up to 3,000 m is, from an ophthalmological perspective, safe for both adults and children.

## Data Availability

The raw data supporting the conclusion of this article will be made available by the authors, without undue reservation.
