# Supplementary material for: Retinal blood vessel diameters in children and adults exposed to a simulated altitude of 3,000 m
Source: Front Physiol. 2023 Feb 28;14:1026987. doi: 10.3389/fphys.2023.1026987 (PMC10011172; doi:10.3389/fphys.2023.1026987)
Supplement: Supplementary file 1 [file Table1.DOCX]

**Supplementary Table:** High-altitude (≥3000 m) ski resorts in North America and Europe (Abbreviation: Δ altitude – the difference between peak and base altitude).

| **#** | **Ski Resort** | **Country** | **Base altitude (m)** | **Peak altitude (m)** | **Δ altitude (m)** |
| --- | --- | --- | --- | --- | --- |
| 1 | Breckenridge | Colorado, USA | 2926 | 3914 | 988 |
| 2 | Zermatt/​Breuil-Cervinia/​Valtournenche – Matterhorn | Switzerland and Italy | 1562 | 3899 | 2337 |
| 3 | Loveland | Colorado, USA | 3245 | 3871 | 626 |
| 4 | Aiguille du Midi (Chamonix) | France | 1035 | 3842 | 2807 |
| 5 | Telluride | Colorado, USA | 2659 | 3815 | 1156 |
| 6 | Snowmass | Colorado, USA | 2473 | 3813 | 1340 |
| 7 | Taos | New Mexico, USA | 2805 | 3795 | 990 |
| 8 | Arapahoe Basin | Colorado, USA | 3286 | 3790 | 504 |
| 9 | Silverton Mountain | Colorado, USA | 3147 | 3790 | 643 |
| 10 | Copper Mountain | Colorado, USA | 2926 | 3767 | 841 |
| 11 | Santa Fé | New Mexico, USA | 3154 | 3679 | 525 |
| 12 | Winter Park Resort | Colorado, USA | 2743 | 3676 | 933 |
| 13 | Keystone | Colorado, USA | 2835 | 3651 | 816 |
| 14 | Monarch | Colorado, USA | 3290 | 3630 | 340 |
| 15 | Wolf Creek | Colorado, USA | 3139 | 3628 | 489 |
| 16 | Crested Butte | Colorado, USA | 2774 | 3620 | 846 |
| 17 | Saas-Fee | Switzerland | 1800 | 3573 | 1773 |
| 18 | Cooper | Colorado, USA | 3200 | 3566 | 366 |
| 19 | Aspen Highlands | Colorado, USA | 2451 | 3559 | 1108 |
| 20 | La Grave – La Meije | France | 1470 | 3534 | 2064 |
| 21 | Les 2 Alpes | France | 1280 | 3523 | 2243 |
| 22 | Arizona Snowbowl | Arizona, USA | 2804 | 3505 | 701 |
| 23 | Beaver Creek | Colorado, USA | 2255 | 3488 | 1233 |
| 24 | Apache | New Mexico, USA | 2926 | 3475 | 549 |
| 25 | Monte Bianco - Courmayeur | Italy | 1370 | 3466 | 2096 |
| 26 | Tignes/​Val d'Isère | France | 1550 | 3456 | 1906 |
| 27 | Passo dello Stelvio (Stelvio Pass) | Italy | 2760 | 3450 | 690 |
| 28 | Pitztal Glacier (Pitztaler Gletscher) | Austria | 2685 | 3440 | 755 |
| 29 | Vail | Colorado, USA | 2457 | 3433 | 976 |
| 30 | Aspen Mountain | Colorado, USA | 2422 | 3418 | 996 |
| 31 | Echo Mountain Park – Idaho Springs | Colorado, USA | 3200 | 3401 | 201 |
| 32 | Big Sky Resort | Montana, USA | 2072 | 3398 | 1326 |
| 33 | Mammoth Mountain | California, USA | 2424 | 3369 | 945 |
| 34 | Snowbird | Utah, USA | 2365 | 3353 | 988 |
| 35 | Erciyes – Kayseri | Turkey | 2088 | 3346 | 1258 |
| 36 | Sölden | Austria | 1350 | 3340 | 1990 |
| 37 | 4 Vallées – Verbier/​La Tzoumaz/​Nendaz/​Veysonnaz/​Thyon | Switzerland | 821 | 3330 | 2509 |
| 38 | Alpe d'Huez | France | 1125 | 3330 | 2205 |
| 39 | Sunrise Park | Arizona, USA | 2853 | 3330 | 477 |
| 40 | Brian Head | Utah, USA | 2926 | 3328 | 402 |
| 41 | Beartooth Basin | Wyoming, USA | 3017 | 3322 | 305 |
| 42 | Corvatsch/​Furtschellas | Switzerland | 1797 | 3303 | 1506 |
| 43 | Purgatory - Durango | Colorado, USA | 2680 | 3299 | 619 |
| 44 | Eldora Mountain | Colorado, USA | 2804 | 3292 | 488 |
| 45 | Sierra Nevada – Pradollano | Spain | 2100 | 3282 | 1182 |
| 46 | Gudauri | Georgia | 1993 | 3276 | 1283 |
| 47 | Alagna Valsesia/​Gressoney-La-Trinité/​Champoluc/​Frachey (Monterosa Ski) | Italy | 1212 | 3275 | 2063 |
| 48 | Arabba/​Marmolada | Italy | 1446 | 3269 | 1823 |
| 49 | Angel Fire | New Mexico, USA | 2591 | 3256 | 665 |
| 50 | Hintertux Glacier (Hintertuxer Gletscher) | Austria | 1500 | 3250 | 1750 |
| 51 | Sulden am Ortler (Solda all'Ortles) | Italy | 1900 | 3250 | 1350 |
| 52 | Les 3 Vallées – Val Thorens/​Les Menuires/​Méribel/​Courchevel | France | 1100 | 3230 | 2130 |
| 53 | Eagle Point | Utah, USA | 2773 | 3230 | 457 |
| 54 | Les Arcs/​Peisey-Vallandry (Paradiski) | France | 1200 | 3226 | 2026 |
| 55 | Steamboat | Colorado, USA | 2103 | 3221 | 1118 |
| 56 | Alta | Utah, USA | 2560 | 3215 | 655 |
| 57 | Stubai Glacier (Stubaier Gletscher) | Austria | 1697 | 3212 | 1515 |
| 58 | Val Senales Glacier (Schnalstaler Gletscher) | Italy | 2011 | 3212 | 1201 |
| 59 | Hohsaas – Saas-Grund | Switzerland | 1559 | 3200 | 1641 |
| 60 | Brighton | Utah, USA | 2668 | 3200 | 532 |
| 61 | Jackson Hole | Wyoming, USA | 1924 | 3185 | 1261 |
| 62 | Pajarito Mountain | New Mexico, USA | 2743 | 3182 | 439 |
| 63 | Palandöken (Ejder 3200 World Ski Center) | Turkey | 2200 | 3176 | 946 |
| 64 | La Plagne (Paradiski) | France | 1207 | 3167 | 1960 |
| 65 | Tetnuldi - Mestia | Georgia | 2265 | 3160 | 985 |
| 66 |  | Turkey | 2200 | 3140 | 940 |
| 67 | Sandia Peak | New Mexico, USA | 2630 | 3140 | 510 |
| 68 | Red River | New Mexico, USA | 2640 | 3135 | 495 |
| 69 | Moelltal Glacier (Mölltaler Gletscher) | Austria | 2108 | 3122 | 1014 |
| 70 | Belalp – Blatten | Switzerland | 1322 | 3118 | 1796 |
| 71 | Kaunertal Glacier (Kaunertaler Gletscher) | Austria | 2150 | 3113 | 963 |
| 72 | Lauchernalp – Lötschental | Switzerland | 1403 | 3091 | 1688 |
| 73 | June Mountain | California, USA | 2288 | 3075 | 787 |
| 74 | Heavenly | California, USA | 2001 | 3060 | 1059 |
| 75 | Park City | Utah, USA | 2080 | 3049 | 969 |
| 76 | Grand Targhee | Wyoming, USA | 2310 | 3048 | 738 |
| 77 | Gurgl – Obergurgl-Hochgurgl | Austria | 1793 | 3030 | 1237 |
| 78 | Kitzsteinhorn/​Maiskogel – Kaprun | Austria | 768 | 3029 | 2261 |
| 79 | St. Moritz – Corviglia | Switzerland | 1720 | 3022 | 1302 |
| 80 | Titlis – Engelberg | Switzerland | 1003 | 3020 | 2017 |
| 81 | Laax/​Flims/​Falera | Switzerland | 1100 | 3018 | 1918 |
| 82 | Buttermilk Mountain | Colorado, USA | 2399 | 3018 | 619 |
| 83 | Glacier 3000 – Les Diablerets | Switzerland | 1343 | 3016 | 1673 |
| 84 | Sunlight Mountain | Colorado, USA | 2403 | 3015 | 612 |
| 85 | Bormio – Cima Bianca | Italy | 1225 | 3012 | 1787 |
| 86 | Diavolezza/​Lagalb | Switzerland | 2091 | 3006 | 915 |
| 87 | Yellowstone Club | Montana, USA | 2182 | 3005 | 823 |
| 88 | Pejo 3000 | Italy | 1400 | 3000 | 1600 |
| 89 | Ponte di Legno/​Tonale/​Presena Glacier/​Temù (Pontedilegno-Tonale) | Italy | 1121 | 3000 | 1879 |
| 90 | Solitude | Utah, USA | 2434 | 3000 | 566 |
| 91 | Powderhorn | Colorado, USA | 2499 | 3000 | 501 |
| 92 | Bonneval sur Arc | France | 1850 | 3000 | 1150 |
| 93 | Macugnaga | Italy | 1327 | 3000 | 1673 |
| 94 | Kendall Mountain - Silverton | Colorado, USA | 2840 | 3000 | 160 |
